# MK571 inhibits phase-2 conjugation of flavonols by Caco-2/TC7 cells, but does not specifically inhibit their apical efflux^☆^

**DOI:** 10.1016/j.bcp.2015.03.005

**Published:** 2015-06-01

**Authors:** Robert D. Barrington, Paul W. Needs, Gary Williamson, Paul A. Kroon

**Affiliations:** a Institute of Food Research, Norwich Research Park, Norwich NR4 7UA, UK; b School of Food Science and Nutrition, University of Leeds, Leeds, LS2 9JT, UK

**Keywords:** Mrp2, multidrug resistance protein-2, Caco2, cancer of the colon cells, MDCKII, Madin–Darby canine kidney cells, ABC, ATP-binding cassette, Ap, apical, Bl, basolateral, HPLC, high pressure liquid chromatography, Q, quercetin, K, kaempferol, G, galangin, GlcA, glucuronide, S, sulphate, K-S-GlcA, kaempferol-sulfoglucuronide, K-4′-GlcA, kaempferol-4′-O-glucuronide, K-3-GlcA, kaempferol-3-O-glucuronide, K-7-GlcA, kaempferol-7-O-glucuronide, K-S, kaempferol-sulphate, Q-7-GlcA, quercetin-7-O-glucuronide, Q-3-GlcA, quercetin-3-O-glucuronide, Q-3′-GlcA, quercetin-3′-O-glucuronide, Q-4′-GlcA, quercetin-4′-O-glucuronide, Q-7-S, quercetin-7-O-sulphate, Q-3′-S, quercetin-3′-O-sulphate, G-5-GlcA, galangin-5-O-glucuronide, G-3-GlcA, galangin-3-O-glucuronide, G-7-GlcA, galangin-7-O-glucuronide, G-S, galangin sulphate, Flavonols, Flavonoids, MK571, Caco-2/TC7, Multidrug resistance protein 2, Phase-2 conjugation

## Abstract

MK571 is a multidrug resistance protein-2 (ABCC2, Mrp2) inhibitor and has been widely used to demonstrate the role of Mrp2 in the cellular efflux of drugs, xenobiotics and their conjugates. Numerous reports have described modulation of Caco-2 cellular efflux and transport of flavonoids in the presence of MK571. Since flavonoids are efficiently conjugated by Caco-2/TC7 cells, we investigated the effects of MK571 on the efflux of flavonoid conjugates. The flavonol aglycones kaempferol, quercetin and galangin were efficiently taken up, conjugated and effluxed by Caco-2/TC7 cells. Apically-applied MK571 caused significant reductions in both the apical and basolateral efflux of flavonol conjugates from Caco-2/TC7 monolayers. MK571 did not significantly alter the apical:basolateral efflux ratio for flavonol conjugates, however, which is not consistent with MK571 specifically inhibiting only apical Mrp2. Since MK571 decreased the total amounts of conjugates formed, and increased cellular flavonol aglycone concentrations, we explored the possibility that MK571 also inhibits phase-2 conjugation of flavonols. MK571 dose-dependently inhibited the intracellular biosynthesis of all flavonol glucuronides and sulphates by Caco-2 cells. MK571 significantly inhibited phase-2 conjugation of kaempferol by cell-free extracts of Caco-2, and production of kaempferol-4′-O-glucuronide was competitively inhibited. These data show that MK571, in addition to inhibiting MRP2, is a potential inhibitor of enterocyte phase-2 conjugation.

## Graphical abstract

**Figure:**
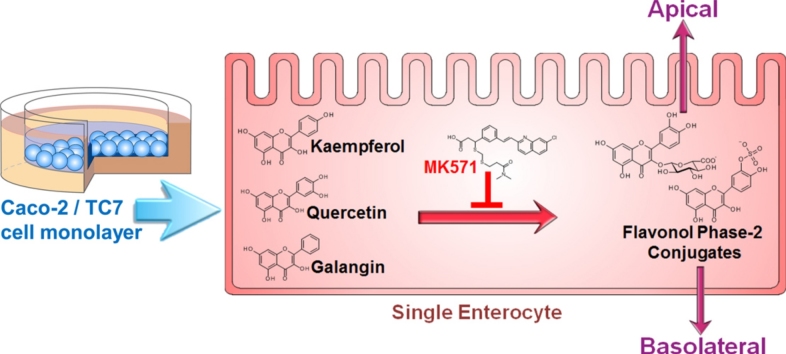

## 1 Introduction

Kaempferol, galangin and quercetin are flavonoids belonging to the sub-class flavonols. Flavonols are secondary plant metabolites present in plant tissues mostly in the form of glycosides. Flavonoids are bioavailable in humans [1–7] and animals [8–11], and upon ingestion, enter the small intestine where luminal hydrolysis of the glycoside bond may occur for β-d-glucosides and β-d-galactosides, by the brush border β-glucosidase lactase-phloridzin hydrolase [12,13]. Following hydrolysis, the flavonoid aglycone can diffuse into the enterocytes [14]. Following cellular uptake of flavonol aglycones, extensive metabolism occurs within enterocytes which leads to the formation of phase-II conjugates, most notably sulphates and glucuronides, as well as methylated and mixed conjugates [15–19]. The metabolism of flavonoids has a major impact on the chemical properties of flavonols (e.g. molecular mass, hydrophobicity) and on their biological activities, including those that would be expected to confer health benefits [20–25]. Conjugation also leads to a substantial increase in hydrophilicity, and the subsequent efflux of flavonoid conjugates from enterocytes occurs via an active transport process [26,27].

Several studies identified Mrp2 as a transporter likely involved in the apical efflux of flavonols based on the use of the Mpr2 inhibitor MK571 to inhibit apical transport of chrysin conjugates [28], quercetin-4′-β-glucoside [29], or cause cellular accumulation of quercetin-4′-β-glucoside [30,31]. In addition, quercetin glucuronides interacted with Mrp2 based on *in silico* calculations and on experimental estimation of interactions of quercetin glucuronides with Mrp2 expressed in ABCC2-overexpressing baculovirus-infected Sf9 cells [32]. On the other hand, [33] used Madin–Darby canine kidney-2 (MDCK-2) cells transfected with human Mrp2 and reported no difference in the efflux of quercetin conjugates when compared to control cells, suggesting Mrp2 is not involved in the efflux of quercetin conjugates. The same authors demonstrated that following intestinal uptake of quercetin, efflux of quercetin glucuronides was no different between Mrp2 deficient and control rats.

The various reports providing evidence that flavonols and their phase-2 conjugates can interact with MRP2 and that treatment with the Mrp2 inhibitor MK571 results in reduced rates of transport/apical efflux are not consistent with evidence provided by the [33] report which concluded that Mrp2 was not involved. We hypothesised that MK571 interferes with flavonol conjugation, noting that if MK571 inhibited phase-2 conjugation of flavonols, then a reduction in apical efflux of these conjugates would be observed. Therefore, we used Caco-2/TC7 cells, which efficiently conjugate flavonols, and investigated the potential for MK571 to influence both the conjugation of flavonols and their efflux from the cells.

## 2 Materials and methods

All cell culture supplies were from Invitrogen, Paisley, UK, unless otherwise stated. Caco-2/TC7 cells were kindly donated by Dr M. Rousset, U178 INSERM, Villejuif, France. Millicell-ERS volt ohmmeter was obtained from Millipore corporation, Massachusetts, USA. Transwell inserts and 12 well plates were Costar brand obtained from Fisher Scientific, Loughborough, UK. The MK571 was purchased from Biomol Research Laboratories, Exeter, UK. Mini protease inhibitor cocktail tablets containing EDTA and perfabloc were purchased from Roche, Welwyn Garden City, UK. Galangin, quercetin and kaempferol were purchased from Extrasynthese, 69727 Genay Cedex, France. Alamethacin from *Trichoderma viride*, uridine 5′-diphosphoglucuronic acid and uridine 5′-diphospho-N-acetyl-glucosamine were purchased from Sigma–Aldrich, Poole, UK. GraFit software was version 4.0 and purchased from Erithacus Software Ltd., East Grinstead, UK. Gemini C18 (150 mm × 2.0 mm i.d., 5 μm) narrow bore HPLC column was purchased from Phenomenex, Macclesfield, UK. Analytical chemistry was performed using ChemStation software on an HP1100 HPLC and single quadrupole MSD SL mass spectrometer from Agilent, Manchester, UK. All solvents and other reagents of analytical grade were obtained from Sigma–Aldrich, Poole, UK.

### 2.1 Cell culture

Cells were grown at 37 °C in a humidified incubator with 5% CO_2_ in Dulbecco Modified Eagle's Medium (DMEM) containing 1% non-essential amino acids, 1% l-glutamine, 100 IU/mL penicillin and 100 μg/mL streptomycin, 1 g/L glucose, and supplemented with 20% (v/v) heat inactivated fetal calf serum. Cells were passaged at 80% confluency, and seeded at 2–4 × 10^4^ cells per cm^2^ on 10 cm dishes (growing area 75 cm^2^) or 12-well transwells (growing area of 1.1 cm^2^). Cells were used for experiments when trans epithelial electrical resistance values of wells showed values greater than 180 Ω/cm^2^ using a Millicell-ERS volt ohmmeter, which was typically 21–23 days post confluency. Toxicity of MK571 on Caco-2/TC7 cell monolayers was determined through comparison of pre- and post-loading TEER values of wells incubated with MK571.

### 2.2 Flavonol metabolism and transport experiments, and effects of MK571

Flavonol stocks (quercetin, kaempferol, galangin; 10 mM) were prepared in dimethylsulfoxide (DMSO) and stored at −20 °C. MK571 was dissolved in dimethyl sulfoxide (10 mM final concentration) and stored at −20 °C. Prior to the metabolism or transport experiments, cells were aspirated of media and washed three times with phosphate buffered saline (PBS). To foetal calf serum and phenol red-free DMEM was added ascorbic acid at a final concentration of 100 μM as well as MK571 at the desired concentration. For metabolism experiments, 10 mL of this media was added to the cells in 10 cm dishes. For transport experiments using transwells, 0.6 mL of this media was added to the apical compartment of the transwell and 1 mL was added to the basolateral compartment. Control wells received an equivalent dose of dimethyl sulfoxide as the treatment wells. Cells were incubated at 37 °C for 30 min, after which flavonols dissolved in dimethyl sulfoxide (kaempferol, galangin or quercetin) were added to the media of treatment wells at a final concentration of 40 or 100 μM, while control wells were treated with the dimethyl sulfoxide vehicle alone. Concentrations of quercetin were increased to 100 μM because instability of quercetin in media necessitated higher initial loading to maintain adequate cellular and media concentrations for uptake and metabolism of the flavonol. Control and treatment cells were incubated at 37 °C for the periods indicated in the results.

Following the required incubation period, media and cell fractions were harvested and analysed to identify and quantify flavonol metabolites using HPLC. Media was removed from the apical (0.5 mL) and basolateral (0.5 mL) compartments of transwells and added to 25 μL trifluoroacetic acid and 25 μL HPLC grade acetonitrile. Media from 10 cm dishes (1 mL) was removed and added to 50 μL trifluoroacetic acid and 25 μL HPLC grade acetonitrile. Cells were washed three times with cold (room temperature) phosphate buffered saline solution and scraped free of the dish surface. For transwells, 100 μL MilliQ water was added to the cells and the resulting cell suspension pipetted into 10 μL trifluoroacetic acid and 10 μL HPLC grade acetonitrile. For 10 cm dishes, the resulting cell suspension was pipetted into 25 μL trifluoroacetic acid and 25 μL HPLC grade acetonitrile. Samples were either analysed immediately or otherwise stored at −20 °C prior to HPLC analysis. Flavonols were stable during frozen storage and thawing (no significant losses up to 26 weeks).

### 2.3 Sample preparation and analytical chemistry

Media samples were thawed where necessary and then mixed using a vortex mixer, centrifuged for 10 min at 14,000 × *g* using a micro centrifuge, and the supernatant removed and placed in an HPLC vial for analysis. Cell samples were vortex-mixed and then ultra-sonicated (sonic water bath) for 10 min, vortex mixed again and then centrifuged for 10 min at 14,000 × *g* using a micro-centrifuge, before removal of supernatant for HPLC analysis. Identification and quantification of individual flavonol metabolites was carried out using liquid chromatography–mass spectrometry and nuclear magnetic resonance analyses, as described previously [19]. The quantities of analytes (individual flavonol conjugates or total conjugates) present in the media from 10 cm dishes and apical/basolateral media samples from transwells, and in cell fractions, were calculated from the respective HPLC chromatogram peak areas using standard curves generated with authentic standards where available or similar analytes where standards were not available.

### 2.4 Determination of apical (Ap) to basolateral (Bl) ratios from transport experiments

The amount of each analyte was calculated for each of the apical and basolateral compartments in transwell experiments, and rates were calculated as pmol flavonol conjugates min^−1^ cm^−2^ cells. The apical to basolateral ratio was calculated using the following equation:

$$\text{Ap}:\text{Bl}\;\text{ratio}=\frac{\text{molar}\;\text{quantity}\;\text{of}\;\text{analyte}\;\text{in}\;\text{apical}\;\text{compartment}}{\text{molar}\;\text{quantity}\;\text{of}\;\text{analyte}\;\text{in}\;\text{basolateral}\;\text{compartment}}$$

### 2.5 Cell free assays and enzyme kinetics

Cells were grown until 21 days post confluent and washed and aspirated as described previously. The cells were then scraped into tubes and these tubes kept on ice at all times throughout the preparation procedure. Homogenisation buffer comprised of sodium phosphate buffer (50 mM adjusted to pH 6.5; 20 mL), two mini protease inhibitor cocktail tablets containing EDTA and perfabloc, dithiothreitol (30.8 mg), alamethacin (20 μL of a 22 μg/mL) solution from *T. viride*; and MgCl_2_ (3.5 mg). Cell-free homogenates were prepared by mixing cells with homogenisation buffer (50:50, vol/vol) and vortexing with glass beads in an eppendorf tube three times for 1 min over a 10 min period. To 900 μL of cell free lysate was added 10 μL uridine 5′-diphosphoglucuronic acid (4 mM final concentration) with uridine 5′-diphospho-N-acetyl-glucosamine (dissolved in homogenisation buffer, 2 mM final concentration); 10 μL MK571 (50 mmol/L stock diluted 1:9 with homogenisation buffer) or 10 μL dimethyl sulfoxide blank (diluted 1:9 with homogenisation buffer). The tubes were allowed to warm and once they reached 37 °C, the flavonols were added at the relevant concentration (kaempferol, quercetin or galangin; 10 mmol/L stock in dimethyl sulfoxide diluted 1:1 with homogenisation buffer, plus homogenisation buffer added to create a final volume of 1000 μL) and the tube vortexed briefly. The experiment was stopped by addition of a mixture of 50 μL trifluoracetic acid and 50 μL acetonitrile. Samples were then centrifuged for 10 min at 14,000 × *g*. The supernatant was removed and analysed via HPLC.

### 2.6 Estimation of *K*_*m*_, *V*_max_ and *K*_*i*_ values

Estimates of initial rates (*v*) of formation of flavonol conjugates by UDP-β-d-glucuronosyl transferase (UDP-GT) activity in the Caco2 cell-free extracts were calculated for each concentration of S (S = kaempferol; [S] range 5–150 μM). Reciprocal plots (1/*v* versus 1/S) were generated and examined for linear fit. At concentrations where good linear fit in reciprocal plots was observed, *K*_*m*_ and *V*_max_ values were estimated using the weighted least-squares linear regression algorithm available in the GraFit software [34]. The type of inhibition was determined by plotting 1/*v* versus 1/S for each of the inhibitor (MK571) concentrations tested and observing the location of the intersection of the lines, which also gave estimated values for *K*_*m*_ and *V*_max_. Subsequently, the slopes of the primary reciprocal plots (*y*-axis) were plotted against the concentration of inhibitor ([MK571], *x*-axis) and used to obtain the *K*_*i*_:

Slope (of best fit line) = *K*_*m*_/(*V*_max_ × *K*_*i*_)
*y*-Intercept = *K*_*m*_/*V*_max_
*K*_*i*_ = *y*-intercept/slope

### 2.7 Statistical analyses

All statistical analysis was carried out using GraphPad online software (www.graphpad.com) using an unpaired t-test, with a *P* value of <0.05 as indicating statistical significance. Application of a Shapiro-Wilk test on each group of data was done prior to application of the statistical test of significance, and all of the Shapiro-Wilk tests suggested that the data came from a normal distribution.

## 3 Results

### 3.1 MK571 inhibits the rate of apical efflux of flavonol conjugates from Caco-2/TC7 monolayers

**Fig. 1:**
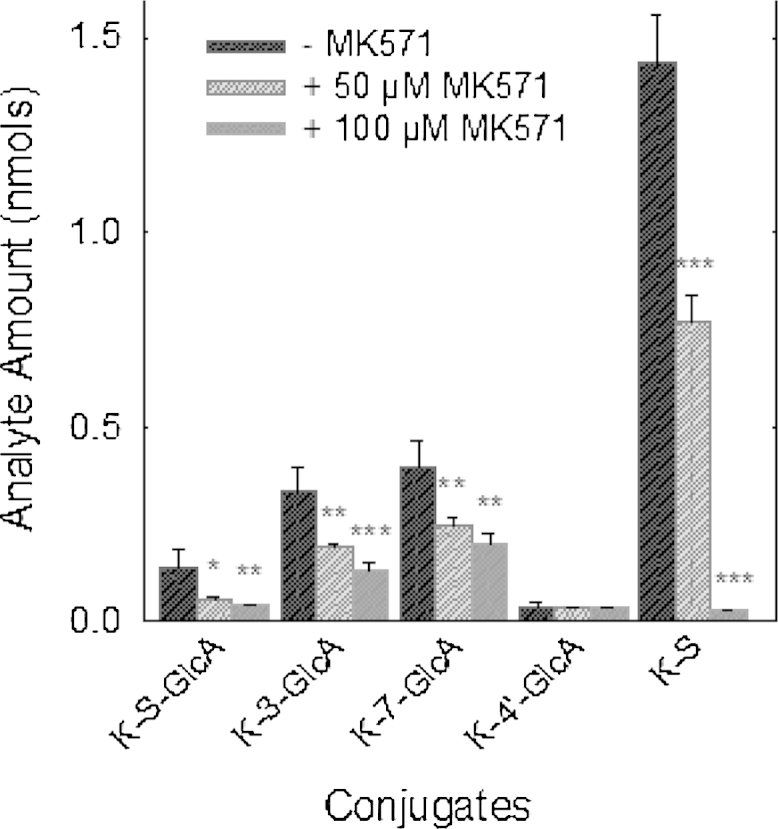
The effect of MK571 on kaempferol conjugate efflux in clonal Caco-2/TC7 cells grown on 12 well transwell monolayers. The kaempferol aglycone (40 μM) was incubated on Caco-2/TC7 monolayers with MK571. After 60 min samples of media were taken from the apical compartments and analysed for kaempferol glucuronide (-GlcA) and sulphate (-S) conjugates. *P < 0.05; **P < 0.01; ***P < 0.001; *n* = 4 for all data points.

**Fig. 2:**
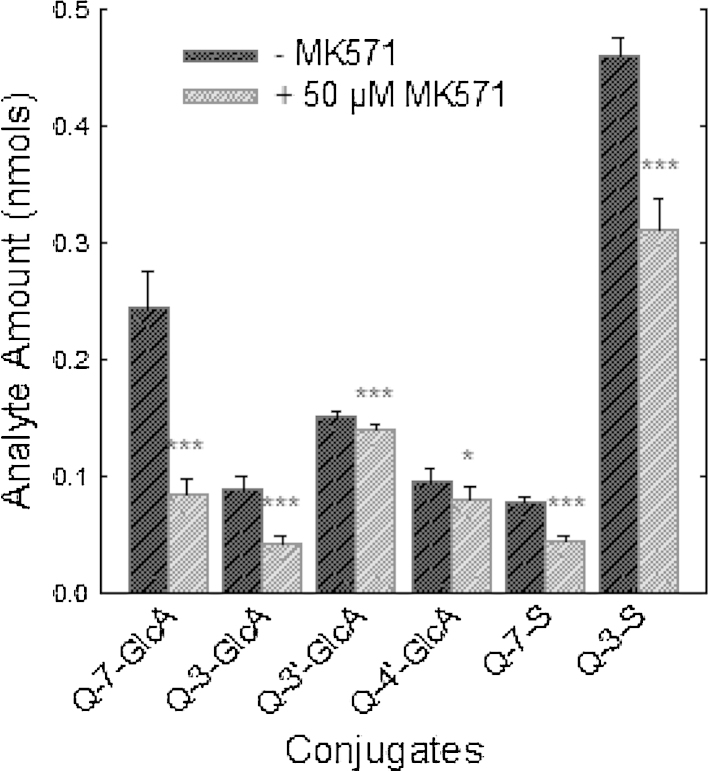
The effect of MK571 on quercetin conjugate efflux in clonal Caco-2/TC7 cells grown on 12 well transwell monolayers. The quercetin aglycone (100 μM) was incubated on Caco-2/TC7 monolayers with MK51. After 60 min samples of media were taken from the apical compartments and analysed using for quercetin glucuronide (-GlcA) and sulphate (-S) conjugates. *P < 0.05; **P < 0.01; ***P < 0.001; *n* = 6 for all data points.

**Fig. 3:**
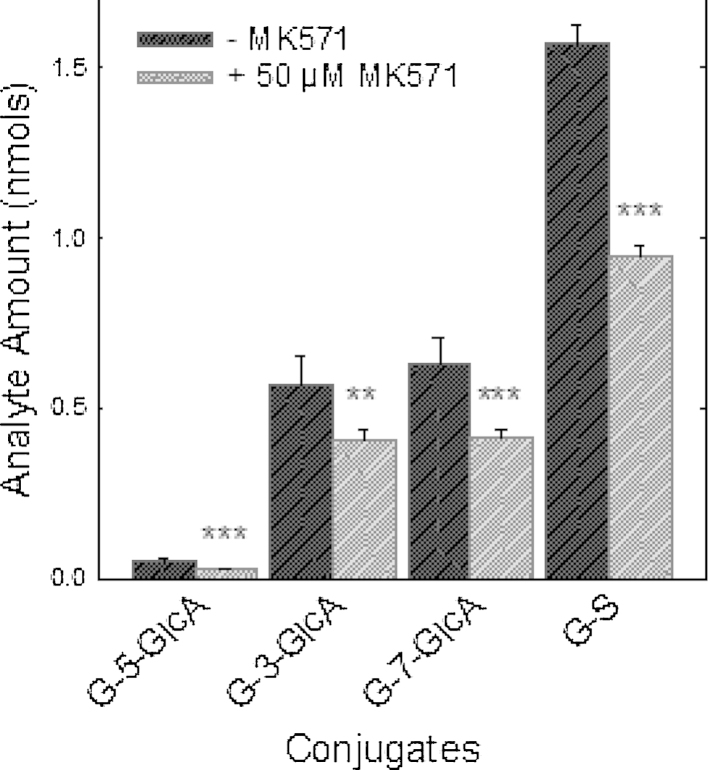
The effect of MK571 on galangin conjugate efflux in clonal Caco-2/TC7 cells grown on 12 well transwell monolayers. The galangin aglycone (40 μM) was incubated on Caco-2/TC7 monolayers with MK51. After 60 min samples of media were taken from the apical compartments and analysed for galangin glucuronide (-GlcA) and sulphate (-S) conjugates. *P < 0.05; **P < 0.01; ***P < 0.001; *n* = 4 for all data points.

The effect of MK571 on the efflux of phase-2 conjugates (sulphates, glucuronides and methylated derivatives) of flavonols (galangin, kaempferol, quercetin) from intestinal epithelial cells was investigated using a differentiated Caco-2/TC7 cell model. When Caco-2/TC7 cell monolayers were incubated with kaempferol in the absence of MK571, kaempferol-sulfo-glucuronide, kaempferol-3-glucuronide, kaempferol-7-glucuronide, kaempferol-4′-glucuronide, kaempferol-3-sulphate and kaempferol-7-sulphate were formed. Details of their structural identification are reported elsewhere [19]. In the presence of MK571 (50 μM), the amount of kaempferol conjugates effluxed to the media was significantly (44%; *P* < 0.001) lower than in the absence of MK571, and the inhibition was dose-dependent (Fig. 1). Similar reductions in the appearance of flavonol phase-2 conjugates in the culture media were observed for the structurally similar flavonols quercetin (penta-hydroxy flavonol) and galangin (trihydroxy flavonol). Compared to vehicle-only control incubations, addition of MK571 (50 μM) to Caco-2/TC7 monolayers resulted in reductions of 41% and 38% in the efflux of quercetin and galangin to the apical compartment, respectively (Figs. 2 and 3). Significant increases in the flavonol aglycone concentrations of the cellular extract accompanied the reductions in conjugates seen in the apical compartments for kaempferol (+182%; *P* < 0.05), galangin (+180%; *P* < 0.001) and quercetin (+119%; *P* < 0.05) with addition of 50 μM MK571, and for kaempferol (+229%; *P* < 0.01) with addition of 100 μM MK571.

### 3.2 MK571 inhibits total flavonol conjugate production by Caco-2/TC7 monolayers

The data presented in Figs. 2 and 3 shows that MK571 treatment causes significant and substantial reductions in the efflux of phase-2 conjugates across the apical membranes of enterocyte monolayers of all three flavonols tested. These data are in keeping with MK571-induced inhibition of the apical efflux transporter Mrp-2. However, the observed MK571-induced reduction in accumulation of flavonol conjugates in the apical media of Caco-2/TC7 monolayers is also consistent with a reduction in the rate of phase-2 metabolite formation. We examined the effects of MK571 on total conjugate production by summing the flavonol conjugates present in the apical, basolateral and cellular compartments at the end of incubations with flavanols in the presence or absence of MK571. The data presented in Table 1 clearly show that for all three flavonols, the presence of MK571 caused significant (*P* < 0.001) reductions in the synthesis of phase-2 conjugates by the cells. Furthermore, the magnitudes of the MK571-induced reductions in flavonol-conjugate production were very similar to the MK571-induced reductions in apical efflux of the flavonol conjugates. Taken together, these observations provide indirect evidence to support the notion that the MK571-induced decrease in apical efflux of flavonol conjugates was caused by MK571-mediated inhibition of flavanol conjugation by the enterocytes. For all flavonols, decreases in the total amounts of metabolites were caused by lower amounts of both glucuronide and sulphate conjugates. Production of total kaempferol glucuronides was reduced by 52.3 and 67.2%, and kaempferol sulphates by 50.8 and 88.4% in the presence of 50 and 100 μM MK571, respectively. Quercetin and galangin glucuronides were similarly decreased by 56.5 and 42.8%, respectively, and quercetin and galangin sulphates by 39.6% and 37.0%, respectively.

**Fig. 4:**
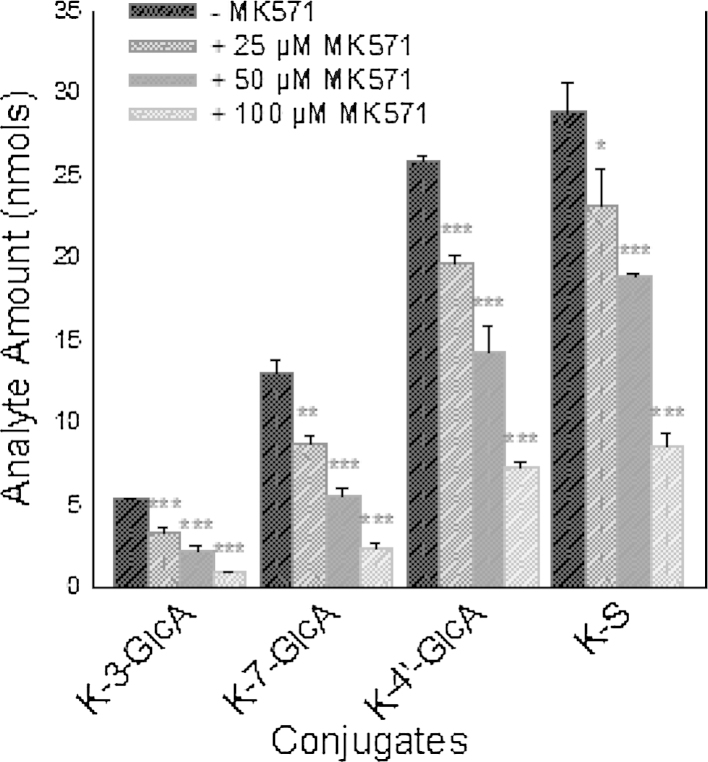
The effect of MK571 on kaempferol metabolism in clonal Caco-2/TC7 cells grown on 10 cm dishes. Kaempferol aglycone (40 μM) was incubated in Caco-2/TC7 cells grown on 10 cm dishes with 25, 50 and 100 μM of MK51. After 60 min samples were taken from the media and analysed for kaempferol glucuronide (-GlcA) and sulphate (-S) conjugates. K-S-GlcA was not detected. *P < 0.05; **P < 0.01; ***P < 0.001; *n* = 3 for all data points.

**Fig. 5:**
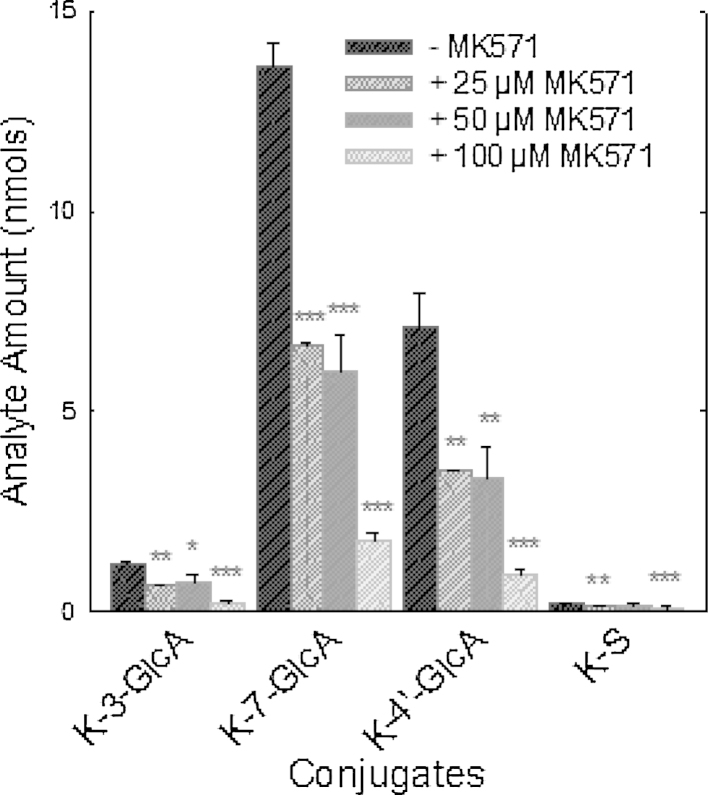
The effect of MK571 on kaempferol metabolism in clonal Caco-2/TC7 cells grown on 10 cm dishes. Kaempferol aglycone was incubated in Caco-2/TC7 cells grown on 10 cm dishes with 25, 50 and 100 μM of MK51. After 60 min samples of cells were taken and analysed for kaempferol glucuronide (-GlcA) and sulphate (-S) conjugates. *P < 0.05; **P < 0.01; ***P < 0.001; *n* = 3 for all data points.

The small surface area of the 12 well transwell inserts made detailed analysis of the cellular content of metabolites difficult. Therefore to assess the effect of MK571 on the conjugation of individual kaempferol metabolites in more detail, kaempferol was added to 10 cm dishes in order to generate larger amounts of metabolites. Addition of MK571 caused significant concentration-dependent reduction in the levels of kaempferol conjugates at 60 min in both the media (Fig. 4) and cellular compartments (Fig. 5).

### 3.3 MK571 does not decrease the apical to basolateral efflux ratio of flavonol glucuronides in transwell monolayers

If MK571 exclusively inhibits apical transport, it would be expected that the apical efflux would be decreased relative to the basolateral efflux, resulting in a decrease in the apical to basolateral efflux ratio. Therefore, in order to determine the degree of apical inhibition, the apical to basolateral efflux ratios of the flavonoid conjugates were measured in the presence and absence of apical MK571. Apical to basolateral ratios for all flavonol glucuronide conjugates showed a dose dependent increase with addition of MK571 (Table 2). Only kaempferol sulphate at the 100 μM concentration and galangin sulphate at the 50 μM concentration showed reductions in the apical to basolateral ratio. In order to further investigate the effect of MK571 on the metabolism of flavonol conjugates, cell free experiments using Caco-2/TC7 homogenates were undertaken. Incubation of the cell-free lysate with galangin and kaempferol resulted in significant reductions in production of kaempferol-3-glucuronide, kaempferol-7-glucuronide, galangin-3-glucuronide and galangin-7-glucuronide, in the presence of MK571 (Table 3).

### 3.4 The effect of MK571 on kaempferol-4′-glucuronide conjugation

To investigate the effect of MK571 on the glucuronidation and sulfation of individual kaempferol conjugates further, a range of concentrations of aglycone were incubated with MK571 and the cell lysate. Addition of MK571 resulted in concentration-dependent reductions in the production of all kaempferol phase-II conjugates. The Lineweaver–Burk reciprocal plots for each of the glucuronidated products of kaempferol (O-β-d-glucuronides conjugated at distinct positions on the flavonol; -4′-, -3- and 7-GlcA) were different. However, substrate inhibition occurred at the higher concentrations of kaempferol, except for kaempferol-4′-glucuronide (K-4′-O-GlcA) where the data gave a reasonable fit to the Michaelis–Menten curve and *K*_*m*_ and *V*_max_ values were easy to estimate. In the presence of increasing concentrations of MK571, the *V*_max_ for synthesis of K-4′-O-GlcA remained unaffected but the *K*_*m*_ was increased (=decreased affinity). This observation is entirely consistent with competitive inhibition of the synthesis of K-4′-O-GlcA by MK571. The estimated *K*_*i*_ for inhibition of the synthesis of K-4′-O-GlcA by MK571 was 19.7 μM.

## 4 Discussion

Efflux of flavonol conjugates back to the lumen of the gut contributes to a reduction in overall absorption of flavonols. Here we show that the addition of the Mrp2 inhibitor MK571 apically to Caco-2/TC7 monolayers resulted in a reduction in the efflux of flavonol conjugates to the apical compartment, as well as decreased quantities inside cells and on the basolateral side. These findings are not consistent with a specific effect of MK571 only on apical transport as reported for many compounds previously. Instead, our observations are more consistent with an inhibitory effect of MK571 on the cellular conjugation of the flavonols, i.e. by inhibition of UDP-glucuronosyl transferases and aryl-sulfatases. In support of this, measurement of the rate of production of flavonol conjugates using a Caco-2/TC7 cell-free extract demonstrated the direct inhibitory effects of MK571 on conjugation production.

Our results show that apical efflux of quercetin, galangin and kaempferol conjugates from Caco-2/TC7 transwell monolayers are reduced by 37.1, 38.5 and 43.2%, respectively, in the presence of 50 μM MK571. In a similar way, chrysin glucuronide and sulphate efflux was inhibited by 71% and 25%, respectively, in the presence of 50 μM MK571 in Caco-2 cells (Walle et al. [28]). Further, efflux of methylated sulphate conjugates of epigallocatechin and sulphate conjugates of epigallocatechin were substantially inhibited in the presence of 50 μM MK71 [35], and apical efflux of epicatechin sulphate conjugates was inhibited by >50% [36]. These studies concluded that Mrp2 played a major role flavonoid conjugate efflux, supported by inhibition studies using Mrp2-over-expressing cells and *in silico* modelling (Williamson et al. [32]). Using more detailed analysis, here we show that in addition to these inhibitory activities, inhibition of phase 2 conjugation is also important, and may even predominate for flavonols when used in a Caco-2/TC-7 monolayer model of intestinal absorption. The TC7 clone of Caco-2 cells is much more efficient than “conventional” clones at phase 2 conjugation including higher expression of all SULTs [37] and of some UGT isoforms [38]. Therefore, the activity of MK571 on phase 2 conjugating enzymes may be shifted in conjugation-competent cells, and this interaction is potentially missed in the other Caco-2 lines. Apigenin glucuronidation was inhibited by 30–40% in the presence of 50 μM MK571, whereas sulfation was not inhibited [39].

Examination of kinetics using a range of kaempferol and MK571 concentrations with Caco-2/TC7 cell free lysate showed a MK571-concentration-dependent reduction in the production of all kaempferol phase-II conjugates, with apparent substrate inhibition at high concentrations of kaempferol. The exception was for kaempferol-4′-glucuronide, where the data gave a reasonable fit to the Michaelis–Menten curve, with competitive inhibition by MK571. This is the first report of competitive inhibition of MK571 on the glucuronidation of flavonoids and the first time it has been demonstrated that MK571 causes inhibition of both glucuronsyltransferase and aryl-sulfatase enzymes. The apparent substrate inhibition observed in cell-free extract incubations could also account for the inhibition of glucuronidation and sulfation seen in Caco-2/TC7 transwell experiments. The observation that cellular concentrations of flavonol aglycone increased with addition of MK571 is consistent with this line of reasoning, and substrate inhibition by the parent aglycone accumulating in the cells may therefore account for some of the inhibitory effect on flavonoid conjugation. Nevertheless, the lack of effect of MK571 treatment on the apical:basolateral ratios for any of the flavonol conjugates confirms that MK571 is not specifically affecting efflux transport of the flavonol conjugates (and in particular MRP-2 mediated efflux to the apical compartment) and that its major effect is to reduce the rate of conjugation of flavonols. Although the potential inhibitory effects of MK571 on individual UGT isoforms were not tested in this study, this is something that could be explored in future work.

Mrp2 is not essential for the apical efflux of flavonol conjugates in some cases. Using Mrp2 deficient rats, luminal efflux of quercetin glucuronides and plasma levels of quercetin did not differ between control rats, suggesting that another transporter was responsible for the effect [33]. The breast cancer resistance protein 1 (Bcrp1; ABCC3) may be involved in apical efflux of quercetin from enterocytes as shown using Mrp2 deficient rats where the Bcrp1 inhibitor FTC increased total plasma quercetin and isorhamnetin concentrations to twice that of controls [33], and the role of Bcrp1 was supported by apical efflux of quercetin in mouse Bcrp1 transfected (MDCK)II cell monolayers but with no directional transport of quercetin in either human Mrp2 transfected MDCKII or parental (MDCK)II monolayers. However, no conjugates could be detected in either the apical or basolateral compartment of the transfected or parental (MDCK)II cells, and so only efflux of quercetin aglycone was observed, which suggests that the cells did not contain UDP-glucuronosyl transferase nor aryl-sulfatase activity.

The metabolism by intestinal cells involves uptake of the aglycone, conjugation by phase 2 enzymes, and efflux by Mrp2, Bcrp1 or other transporters. In cell models, the ratio of activities of these three steps will vary between different cell lines and different clones. In TC7 cells, phase 2 conjugation is increased relative to other cells, and hence this step is increased in importance as a target for an inhibitor. It is also possible that the effects of MK571 on phase-2 conjugation described here on Caco-2/TC7 cells are not replicated in other cell types, for example because the effects are specific to the enzymes expressed in CaCo2 cells or the small intestine, and other isoforms of the enzymes may not be affected in the same way. Further research to establish the effects of MK571 on phase-2 conjugation in other cell types and the characterisation of the susceptibility of the various isoforms of UDP-glucuronosyltransferase and aryl-sulfatase is warranted. MK571 may cause inhibition of glucuronidation and apical transport at the concentrations previously used in transport studies (50 μM), but at lower concentrations (10 μM), the effects on glucuronidation may be attenuated somewhat [39]. Further, the identification of Mrp2 in the transport of flavonoid aglycones and glycosides based on inhibition studies using MK571 in Caco-2 transwell monolayer studies is complicated because MK571 has been reported to show inhibition of Mrp1 [40], Mrp2, [41], Mrp4 [42], Mrp5 [42], and organic anion transporters (OAT) [41,43] and may therefore have broad efflux pump activity. While Mrp1 is not extensively expressed in Caco-2 cells [28], it cannot be discounted that MK571 is able to inhibit the activity of multidrug resistant proteins other than Mrp2 in Caco-2 cells. In addition, Bcrp1 has been shown to be responsible for efflux of some other flavonoids such as hesperetin conjugates [44].

In conclusion, we have shown that conjugation of kaempferol, quercetin and galangin in Caco-2/TC7 cells is inhibited in the presence of the Mrp2 inhibitor MK571. Such inhibition may be due to competitive inhibition of the UGT enzyme by MK571 or may result from substrate inhibition by the flavonol aglycone. This inhibition seems to dominate the metabolic pathway and diminish the significance of inhibition of Mrp2, the classical target for MK571. The data presented here suggest that the role of Mrp2 in the efflux of flavonol conjugates may have been overestimated where effects on conjugation have not been taken into account.

## Authorship contributions

*Participated in research design*: Barrington, Kroon, Williamson.
*Conducted experiments*: Barrington, Needs.
*Performed data analysis*: Barrington, Kroon.
*Wrote or contributed to the writing of the manuscript*: Barrington, Kroon, Williamson.

## Figures and Tables

**Table 1:** The effect of MK571 on flavonol conjugation using 21-day post-confluent Caco-2/TC7 cells grown on 12 well transwell inserts. MK571 was added to the apical media of treatment wells at concentrations of 50 or 100 μM. Treatment and control (no MK571) wells were incubated with flavonol (quercetin, kaempferol, galangin) for 60 min after which apical and basolateral media and the cell fraction analysed for flavonols and flavonol glucuronides (-GlcA) and sulphates (-S) using HPLC. Data provided are rates (nmol/h); *n* = 4 for kaempferol (incubation with 40 μM aglycone), *n* = 6 for quercetin (incubation with 100 μM aglycone), *n* = 4 for galangin (incubation with 40 μM aglycone); kaempferol conjugates detected were K-S-GlcA, K-4′-GlcA, K-3-GlcA, K-7-GlcA, K-S; quercetin conjugates detected were Q-7-GlcA, Q-3-GlcA, Q-3′-GlcA, Q-4′-GlcA, Q-7-S, Q-3-S; galangin metabolites detected were G-5-GlcA, G-3-GlcA, G-7-GlcA, G-S.

	Apical	Basolateral	Cellular	Total conjugates
Kaempferol (K)	2.104	3.429	0.005	5.538
K + 50 μM MK571	1.196 (−43.2%)*	1.484 (−56.7%)*	0.004 (−20.0%)	2.684 (−51.5%)*
K + 100 μM MK571	0.382 (−81.9%)***	1.062 (−69.0%)**	0.003 (−24.2%)	1.447 (−73.9%)**
Quercetin (Q)	1.122	2.916	0.004	4.042
Q + 50 μM MK571	0.706 (−37.1%)***	1.275 (−56.3%)***	0.004 (−2.72%)	1.985 (−50.9%)***
Galangin (G)	2.907	4.037	0.006	6.950
G + 50 μM MK571	1.789 (−38.5%)**	2.279 (−43.6%)***	0.004 (−28.7%)***	4.072 (−41.4%)***

* *P* < 0.05.

** *P* < 0.01.

*** *P* < 0.001.

**Table 2:** The effect of MK571 on the apical to basolateral ratio of efflux of flavonol conjugates by confluent Caco-2/TC7 cells grown on 12-well transwells. MK571 was added to the media of treatment wells at concentrations of 50 or 100 μM. Treatment and control (no MK571) wells were incubated with flavonol (quercetin, kaempferol, galangin) for 60 min after which apical and basolateral media and the cell fraction analysed for flavonols and flavonol glucuronides (-GlcA) and sulphates (-S) using HPLC. All data are rates (pmol/h). *n* = 4 for kaempferol, *n* = 6 for quercetin and galangin. N/D = not determined.

Metabolite	Ap:Bl ratio (No MK571)	Ap:Bl ratio (50 μM MK571)	Ap:Bl ratio (100 μM MK571)
K-S-GlcA	0.59 (±0.174)	0.42 (±0.121)	0.43 (±0.028)
K-3-GlcA	0.22 (±0.040)	0.33 (±0.057)*	0.35 (±0.063)**
K-7-GlcA	0.28 (±0.044)	0.35 (±0.063)	0.44 (±0.021)**
K-4′-GlcA	0.26 (±0.066)	0.33 (±0.080)	0.43 (±0.018)*
K-S	3.02 (±0.230)	3.92 (±0.646)	0.14 (±0.006)***
Q-7-GlcA	0.18 (±0.024)	0.20 (±0.019)	N/D
Q-3-GlcA	0.17 (±0.021)	0.23 (±0.023)***	N/D
Q-3′-GlcA	100% apical	100% apical	N/D
Q-4′-GlcA	0.23 (±0.034)	0.25 (±0.035)	N/D
Q-7-S	1.37 (±0.095)	1.50 (±0.134)	N/D
Q-3-S	0.84 (±0.086)	1.01 (±0.191)	N/D
G-5-GlcA	0.19 (±0.020)	0.30 (±0.024)***	N/D
G-3-GlcA	0.24 (±0.033)	0.35 (±0.091)*	N/D
G-7-GlcA	0.54 (±0.058)	0.51 (±0.036)	N/D
G-S	13.6 (±1.440)	9.10 (±0.783)***	N/D

* *P* < 0.05.

** *P* < 0.01.

*** *P* < 0.001.

**Table 3:** The effect of MK571 on the rate of flavonol conjugate production by cell free lysate using 21 days post-confluent Caco-2/TC7 cells. Portions of Caco-2/TC7 cell lysate were incubated with kaempferol, quercetin or galangin aglycones (each at 40 μM) in the presence or absence of MK571 (50 μM) and samples taken at various time points and analysed for flavonol glucuronides (-GlcA) and sulphates (-S) using HPLC. All rate data are the slopes (linear best fit) of the plots of amount of product versus time (pmol/h). Parentheses indicate the standard error of the slope; *n* = 3 for all data points.

Metabolite	Rate control	Rate +MK571	Change in rate (%)	*P* value
K-S-GlcA	0.54 (±0.04)	0.34 (±0.02)	−40.0%	0.124
K-3-GlcA	1.73 (±0.16)	1.40 (±0.08)**	−19.3%	0.007
K-7-GlcA	1.12 (±0.09)	0.69 (±0.02)***	−38.2%	0.000
Q-7-GlcA	1.89 (±0.16)	1.29 (±0.26)	−31.8%	0.299
Q-3-GlcA	1.05 (±0.09)	0.83 (±0.14)	−21.8%	0.398
Q-3′-GlcA	2.09 (±0.12)	2.62 (±0.24)	+25.6%	0.291
Q-4′-GlcA	0.25 (±0.01)	0.28 (±0.03)	+12.1%	0.493
G-3-GlcA	68.8 (±6.12)	25.3 (±2.30)**	−63.2%	0.003
G-7-GlcA	36.5 (±1.49)	19.8 (±1.63)**	−45.7%	0.002

^*^P < 0.05.

** *P* < 0.01.

*** *P* < 0.001.
